# Obesity is associated with increased seminal insulin and leptin alongside reduced fertility parameters in a controlled male cohort

**DOI:** 10.1186/1477-7827-12-34

**Published:** 2014-05-07

**Authors:** Kristian Leisegang, Patrick JD Bouic, Roelof Menkveld, Ralf R Henkel

**Affiliations:** 1 Department of Medical Biosciences, University of the Western Cape, Private Bag X17, Bellville 7535, South Africa; 2 School of Natural Medicine, University of the Western Cape, Private Bag X17, Bellville 7535, South Africa; 3 Division of Medical Microbiology, Department of Pathology, Stellenbosch University & Tygerberg Academic Hospital, Parow Valley, Cape Town 7500, South Africa; 4 Department of Obstetrics and Gynaecology, Stellenbosch University & Tygerberg Academic Hospital, Parow Valley, Cape Town 7500, South Africa

**Keywords:** Obesity, Semen, Insulin, Leptin, Glucose, Male fertility

## Abstract

**Background:**

Obesity appears to be associated with male reproductive dysfunction and infertility, although this has been inconsistent and inconclusive. Insulin and leptin are known mediators and modulators of the hypothalamus-pituitary-testes axis, contributing to the regulation of male reproductive potential and overall wellbeing. These hormones are also present in semen influencing sperm functions. Although abdominal obesity is closely associated with insulin resistance (hyperinsulinaemia), hyperleptinaemia and glucose dysfunction, changes in seminal plasma concentrations of insulin, leptin and glucose in obese males has not previously been investigated.

**Methods:**

This small case controlled study assessed serum and seminal concentrations of insulin, leptin and glucose in obese (BMI > =30; n = 23) and non-obese (BMI < 30; n = 19) males. Following a detailed medical history and examination, participants meeting the inclusion criteria were entered for data analysis. Body parameters such as BMI, waist and hip circumference and the waist hip ratio were measured. Serum and semen samples were collected and assayed for insulin, leptin and glucose. Semen samples also underwent a standard semen analysis, with sperm mitochondrial membrane potential (MMP) and DNA fragmentation (DF).

**Results:**

Obesity was associated with increased serum and seminal insulin and leptin, with no significant difference in seminal glucose. Serum and seminal concentrations of insulin and leptin were positively correlated. Furthermore, obesity was associated with decreased sperm concentration, sperm vitality and increased MMP and DF, with a non-significant impact on motility and morphology.

**Conclusions:**

Hyperinsulinaemia and hyperleptinaemia are associated with increased seminal insulin and leptin concentrations, which may negatively impact male reproductive function in obesity. Insulin was also found to be highly concentrated in the seminal plasma of both groups. This data will contribute to the contradictive information available in the literature on the impact of obesity and male reproduction.

## Background

Obesity, defined by the World Health Organisation (WHO) as a body mass index (BMI) ≥ 30 kg/m^2^, is a medical condition of excess body fat negatively influencing morbidity and mortality via non-communicable disease risks [1,2]. Increased abdominal adiposity is closely associated with various metabolic changes such as glucose intolerance, insulin resistance, hyperleptinaemia and inflammation, mediating a complex and poorly understood pathophysiological phenomenon termed the metabolic syndrome [3-5]. In males, obesity and metabolic syndrome is further associated with hypogonadism [5].

Infertility is defined by the World Health Organisation [6] as ‘*the inability of a couple to achieve conception or bring a pregnancy to term after 12 months or more of regular (three times per week), unprotected sexual intercourse*’. Alongside an increased incidence in obesity, infertility is a growing concern affecting up to 15% of couples trying to conceive globally, with approximately 25-50% of cases attributed to the male partner [7]. Although not always a true reflection of male fertility potential, an assessment of sperm quality based on WHO guidelines [6] is normally used to estimate the fertilisation potential of the male partner [8]. This typically includes semen volume, sperm concentration and total sperm count, total and progressive motility, sperm vitality and normal sperm morphology [6,8]. Further functional analysis of spermatozoa, although not routine nor standardised, are considered as important additional markers of male fertility potential [8]. These include mitochondrial membrane potential (MMP) [9] and DNA fragmentation (DF) [10]. A decrease in sperm quality is considered a major reflection of the decreased ability of the male partner to contribute to fertilisation [11].

The effect of BMI and sperm parameters has been reportedly investigated in thousands of scientific studies [12]. Although several pathophysiological mechanisms for this association have been implicated [5,13], the effect of BMI on sperm characteristics remains controversial. Any negative effect of obesity on sperm parameters is not consistent, nor is there a clear dose–response mechanism reported [5,14]. Various studies have shown obesity to be associated with a reduction in sperm count and concentration, motility, vitality, morphology, and/or DNA integrity. In contrast, other researchers have not found similar relationships [5,14]. This is highlighted by two recent meta-analytical reviews that had numerous opposing conclusions [12,15]. Paternal obesity is also associated with reduced live birth rates following assisted reproductive technology [16]. In addition, recent data suggests that paternal genetic health cues may be transmitted to the child, with the mediator mostly likely occurring via the sperm [13]. Further research on potential mechanisms associated with the impact of obesity on male reproductive health is therefore warranted.

Insulin and leptin have been reported as important regulators of male reproduction via modulation of the hypothalamus-pituitary-testes (HPT) axis [17]. Both hormones have direct and indirect influence on gonadotropic releasing hormone (GnRH), LH and FSH centrally, and Leydig and Sertoli cell function locally [17]. Alongside insulin and leptin being present in seminal fluid, both hormones have been demonstrated to be synthesised and secreted by ejaculated spermatozoa, with apparent autocrine regulatory functions [18-20]. This is supplemented with *in vitro* evidence that insulin increases total motility, progressive motility, acrosome reaction and nitric oxide production in human spermatozoa [21].

This pilot study aimed to determine the concentrations of insulin and leptin, in addition to glucose, in the serum and semen of obese men.

## Methods

This study was approved by the Senate Research Committee (SRC) of the University of the Western Cape (UWC), Bellville, South Africa (approved 30 July 2010, registration number: 10/6/14). Participant selection and clinical consultations occurred between July 2011 and August 2012. All participants signed an informed consent form (approved by SRC, UWC) in order to undergo a full medical consultation, clinical examination, sample collection and relevant biochemical testing. Obese and non-obese males between 21 and 50 years of age attending private clinics in the Western Cape region of South Africa were notified of the study via description leaflets. Further participants were recruited via public advertisements based free consultation, examination and laboratory assessments for chronic disease risk factors. There was no active recruitment of participants with infertility as a main complaint; however, participants with couple infertility were not restricted from entering the study if no other exclusion criteria were identified. Furthermore, there was no limitation for inclusion based on nutrition, exercise, education, socio-economic or cultural and ethnic status. Selection bias was reduced as all interested males were only rejected based on the exclusion criteria described below, and potential participants were screened and investigated on a ‘first come first serve’ basis.

### Study design

At the pre-clinical stage, generally done via telephonic or electronic communication, interested participants were only excluded from the study if they had a history of vasectomy, any known reproductive tract pathology (e.g. genital tract infections, prostatitis, epididymitis, etc.), were on any hormonal therapy (e.g. testosterone, insulin, thyroid replacement) in the last six months, if they were hostpitalised or had any surgery in the last six months, had any pre-diagnosed chronic disease (specifically obesity related pathology such as Cushing’s syndrome, hypothyroidism and T2DM) or were on medications associated with increased obesity risk (e.g. antidepressant medications, cortisone, metformin, insulin, etc.) in the last six months. Those on medications for chronic disease risk parameters, such as hypertension, dyslipidaemia and coagulation (such as aspirin) were permitted into the consultation phase. Potential participants with a history of smoking or recreational drug use in the last six months were excluded from the study.

At the clinical stage, following a detailed description of the study and signing of the informed consent, a full standardised medical history and physical examination was conducted by a trained professional. This was followed by serum and semen sample collection and biochemical assessments. If any clinical or biochemical detection of acute or chronic disease was identified, patients were excluded from the study, and were provided with all results and appropriate advice and/or referral. This included clinically apparent reproductive disorders (e.g. varicocele; epididymitis; pelvic pain syndrome). Specific exclusion criteria based on biochemical data included serum glucose of > 7 mmol/L (indicating possible T2DM), azoospermia for any reason and leukocytospermia (defined as > 0.5 × 10^6^/ml) as an objective sign of reproductive tract infection/inflammation.

A trained clinician consulting with the participants recorded their age, body mass index (BMI), waist circumference (WC) and hip circumference (HC). Body weight (Kg) was taken on a digital scale to the first decimal point with patients in underclothes. WC was measured in centimetres around the abdomen at the midpoint between the highest point of the iliac crest and the lowest point of the costal margin. HC was measured in centimetres around the level of the greater trochanter. Both WC and HC were recorded as the mean of three measurements. The waist-to-hip ratio (WHR) was recorded as the WC divided by the HC.

Participants included in data analysis were divided into two groups based on the WHO definitions of obesity [1,2]. Those with a BMI ≥ 30 were placed in the obese group, whereas participants with a BMI of < 30 were placed in the non-obese group. The non-obese group therefore consisted of normal weight (BMI = 18 – 24.9) and overweight (BMI = 25 – 29.9) participants.

### Serum and semen collection

All samples were collected between 07:00 and 10:00. Venous blood samples were collected via venopuncture of superficial vessels in the antecubital fossa or hands by a trained clinician using sodium fluoride and serum separating tubule (SST) vacutainers®. Participants were expected to be fasting for a minimum of 8 hours prior to collection. Appropriate vacutainers were immediately transported to PathCare Laboratories (Pathcare Park, Goodwood, South Africa), a private commercial pathology laboratory servicing clinical practice and research sectors in South Africa, for insulin and glucose analysis using standard methods for clinical practice. The Quantitative Insulin Sensitivity Check Index (QUICKI) was calculated from fasting blood glucose and insulin concentrations [22]. An SST was centrifuged at 5000 × g for 10 minutes, and serum was transferred to cryovials and frozen at -20°C for a maximum of 6 months prior to leptin assays described below.

Semen samples were collection followed serum collection within 30 minutes via masturbation into sterile wide mouthed containers following a minimum of 3 days and maximum of 5 days abstinence from sexual activity or masturbation. Participants were instructed to collect all semen, and report any semen loss. Following semen analysis as described below, remaining sample was centrifuged at 5000 × g for 10 minutes, and seminal fluid was transferred to cryovials and frozen at -20°C for a maximum of 6 months prior to glucose, insulin and leptin assays described below.

### Standard semen analysis

Seminal fluid was left for 60 minutes at room temperature to liquefy. After liquefaction, semen was transferred to a test tube in which ejaculate volume was recorded to the nearest decimal point. Sperm count and motility (progressive and total) was assessed using the Motility/Concentration module of the Sperm Class Analyzer® (SCA) CASA system version 4.1.0.1 (Microptic S.L., Barcelona, Spain). For analysis, a Nikon Eclipse 50i microscope (IMP, Cape Town, South Africa) equipped with phase contrast optics and a heated stage (37°C) was used. Sperm vitality was assessed using the eosin-nigrosin staining technique [6]. Morphology was assessed by the preparation of a smear and the application of the Papanicoloaou staining method as outlined by WHO [6], and determined by one person (RM) according to strict criteria as described by Menkveld and colleagues [23]. Leukocytes concentration was determined using the peroxidase staining technique as described by Politch and colleagues [24].

### Functional sperm parameters

Spermatozoal mitochondrial membrane potential (MMP) was assessed as described previously [25] using a Zeiss fluorescence microscope (Oberkochen, Germany) for analysis after staining sperm with DePsipher staining kit (R&D Systems Inc., Minneapolis, MN, USA) used as a mitochondrial marker. In brief, semen was diluted 1:5 ratio with human tubal fluid medium (HTFM) prepared according to the method outlined by Quinn and colleagues [26], supplemented with 10 mg/ml Human Serum Albumin (HSA) (Sigma-Aldrich, St. Louis, MO, USA), and centrifuged for 10 minutes at 500 × g. The supernant was discarded, the pellet re-suspended in DePsipher staining solution and incubated for 20 minutes at 37°C in the dark. The DePsipher sperm suspension was then centrifuged at 500 × g, the supernatant was discarded, and the pellet re-suspended in 100 μl pre-warmed 1X reaction buffer. The cells were observed immediately with fluorescence microscopy at 1000-times magnification. Sperm exhibiting a green fluorescence within their mid pieces were regarded as having disturbed MMP, while those sperm showing red fluorescence were regarded as having intact MMP. The percentage of sperm with disturbed MMP was calculated.

Spermatozoa DNA fragmentation (DF) was assessed by the DeadEnd^™^ Colorimetric TUNEL (terminal deoxynucleotidyl transferase dUTP nick end labelling) System (Promega Corp., Madison, WI, USA) assay according to Henkel and colleagues [27], which end labels the fragmented DNA of apoptotic cells. A sample of liquefied semen was diluted in a 1:5 ratio with HTFM/BSA and centrifuged for 10 minutes at 500 × g. The pellet was re-suspended in PBS (Oxoid, Basingstoke, Hampshire, UK). A smear on a Superfrost® slide (Mentzel, Braunschweig, Germany) was made and allowed to air dry and accumulated for future analysis. All slides were analysed within 6 weeks of preparation. Prepared slides were fixed in 4% methanol-free formaldehyde (Sigma-Aldrich, St. Louis, MO, USA) in PBS for 25 minutes at 4°C. Slides were washed in fresh PBS for 5 minutes at room temperature, then sperm cells permeabilized in 0.2% Triton X-100 (Sigma-Aldrich, St. Louis, MO, USA) in PBS for 5 minutes. After adequate rinsing of slides in fresh PBS, cells were allowed to equilibrate using the equilibration buffer (100 μl added to each slide) for 10 minutes. Slides were blotted around the equlibrilated areas and 20 μl TdT incubation buffer was added to an area of 5 cm^2^ and covered with plastic slips. This was incubated in the dark at 37°C for 60 minutes and terminated using SSC diluted appropriately with deionised water for 15 minutes. The slides were washed in fresh PBS at room temperature 5 times for 5 minutes each, before draining excess water. Immediately following washing, DNA fragmentation was assessed by manual counting done using a Zeiss fluorescence microscope (Oberkochen, Germany). A minimum of 100 (those with poor sperm count) and maximum of 200 spermatozoa were counted on each slide and the results expressed as a percentage of cells showing green fluorescence indicating fragmented DNA (TUNEL-positive cells).

### Seminal insulin

Seminal insulin was assayed using the Human Insulin ELISA Kit (RayBiotech, Inc., Norcross, Georgia, USA). This is an *in-vitro* ELISA based assay for the quantitative measurement of insulin. All reagents and frozen seminal fluid samples were thawed and brought to room temperature for analysis. The lower quantitative limit of the ELISA kit is 4 μIU/ml, with an intra- and inter-assay coefficients of variation (CV) of <10% and <12% respectively. All samples were assayed in duplicate, with the mean value recorded for data analysis. Samples were assayed on an ELISA reader obtained from BioTek (Winooski, VT, USA).

### Seminal glucose

Seminal glucose was assayed using the Glucose HK Assay Kit (Sigma-Aldrich, St. Louis, MO, USA). This is an *in-vitro* ELISA based assay for the quantitative measurement of glucose. All reagents and seminal fluid samples were thawed and brought to room temperature for analysis. The CV of the kit based on correspondence with the supplier is 2.0%. If the duplicate samples are within 2% of each other, these were considered accurate and the mean value recorded for data analysis. All samples were assayed in duplicate, with the mean value recorded for data analysis. Samples were assayed on an ELISA reader obtained from BioTek (Winooski, VT, USA).

### Serum and seminal leptin

Serum and seminal leptin was assayed using the Human Leptin ELISA Kit (RayBiotech, Inc., Norcross, Georgia, USA). This is an *in-vitro* ELISA-based assay for the quantitative measurement of leptin. All reagents and serum and seminal samples were thawed and brought to room temperature for analysis. The lower quantitative limit is 2 ng/ml, with an intra- and inter-assay CV of <10% and <12% respectively. All samples were assayed in duplicate, with the mean value recorded for data analysis. Samples were assayed on an ELISA reader obtained from BioTek (Winooski, VT, USA).

### Statistical analysis

Statistical analysis was performed using the MedCalc software (Version 12.0; Mariakerke, Belgium). After testing for normal distribution using the Kolmogorov-Smirnov test, appropriate statistical tests, either parametric or non-parametric were performed. Parametric results are presented as mean ± SD, and non-parametric results are presented as median (range). All correlations were done using the Spearman correlation coefficient, with significant correlations reported as r^2^. Fisher’s Exact Test was used in order to determine significant differences between groups based on cohort distributions and potential confounders between the groups. P-value of <0.05 was considered as significant with all statistical analyses.

## Results

For the preclinical telephonic or electronic screening, 48 participants had requested to join the study. All were accepted into the clinical assessment except for three, one due to recent surgical procedure for kidney stones, and the other two due to a history of vasectomy reversals. In total, 45 males were included for assessment and sample collection. Of these, two obese males were further excluded from the data analysis based on serum glucose > 7 mmol/L, and a further non-obese male participant was excluded due to leukocytospermia. Therefore, a total of 42 male participants were included in the study for data analysis, and were divided into a non-obese (nOb) group (n = 19) and an obese (Ob) group (n = 23).

Participant distributions and potential confounders are detailed in Table 1. The majority of participants in the cohort, and within each group, were Caucasian (71.4%), with 23.8% coloured (described as mixed ethnic origin with ancestry from Europe, Asia and various local tribes such as Khoisan that is unique to Southern Africa, and particularly in the Western Cape region) and 4.8% black participants. No Asians were in the cohort. More participants in the Ob group were on medications related to modification of risk factors for type-2 Diabetes Mellitus and cardiovascular disease (hypertension; cholesterol; COX-inhibitors), however, there was no significant differences between the groups (Table 1).

**Table 1:** Details of the cohort distributions and potential confounders with comparisons between the groups

	**Cohort**	**Non-obese**	**Obese**	**P-value**
	**(n = 42)**	**(n = 19)**	**(n = 23)**	
**Age distribution (years):**				
21–24	2.4	5.3	0	0.465
25–29	11.9	5.3	17.4	0.376
30–34	23.8	36.8	13.0	0.291
35–39	28.5	31.6	26.1	1.000
40–44	16.7	10.6	21.7	0.682
45-50	16.7	10.6	21.7	0.682
**BMI:**				
18–24.9 (normal weight)	16.7	36.8	-	-
25–29.9 (over-weight)	28.5	63.2	-	-
30–34.9 (obese)	21.4	-	39.1	-
35–39.9 (morbidly obese)	21.4	-	39.1	-
40–44.9 (morbidly obese)	11.9	-	21.7	-
**Demographics:**				
Caucasian	71.4	73.7	69.6	1.000
Coloured*	23.8	15.8	30.4	0.488
Black	4.8	10.6	0	0.221
Asian	-	-	-	-
**Medications:**				
Hypertension	19.0	5.3	30.4	0.122
Cholesterol	14.3	5.3	21.7	0.378
COX inhibitors	7.1	0	13.0	0.251
**Insulin Resistance:**				
(QUICKI < 0.357)	61.9	15.8	100	0.006
**Sperm parameters:**				
Oligozoospermia	26.2	15.8	34.8	0.326
Asthenozoospermia	42.9	31.6	52.2	0.568
Necrozoospermia	61.9	52.6	69.6	0.622
Teratozoospermia	78.6	68.4	86.9	0.645
MMP	50.0	15.8	78.3	0.023
DF	42.9	15.8	65.2	0.043
**Recent history of couple infertility**	21.4	10.6	30.4	0.276

Variables are represented as percentages rounded to the nearest decimal point. Recent history of couple infertility was defined as an inability to achieve a conception with regular sexual intercourse over last 12 months. Sperm parameter definitions were based on WHO guidelines [6]. DF (percentage of sperm with fragmented DNA) = > 25% spermatozoa damaged [9]; percentage of spermatozoa with damaged mitochondria (MMP) = > 36% spermatozoa [28]. P-value was determined using Fisher’s Exact Test. *Couloured racial groups refers to a mixed ethnic origin with ancestry from Europe, Asia and various local tribes such as Khoisan that is unique to Southern African regions.

The mean age of the entire cohort was 36.7 ± 6.7 years (range = 24 – 49). Although the mean age in the Ob group (37.9 ± 7.3) was slightly higher than the nOb group, there was no statistical difference between the groups in terms of age (Table 2). The majority of participants (28.5%) in the cohort were in the 35 – 39 years category, with 23.8% in the 30 – 34 years category (Table 1). There was no significant difference between the groups within each age group category (Table 1). The mean body mass index (BMI) of the cohort was 31.1 ± 6.2 (range = 19 – 44). Distributions for BMI within the cohort are provided in Table 1, with the majority of participants (28.5%) in the cohort being classified as over-weight (BMI 25 – 29.9). As expected, the mean BMI was significantly increased in the Ob group (35.8 ± 4.3) compared to the nOb group (25.5 ± 2.4) (Table 2). Similarly, the mean waist circumference (WC), hip circumference (HC) and waist-to-hip ratio (WHR) were all significantly higher in the Ob group compared to the nOb group. Details of these results are provided in Table 2.

**Table 2:** C**linical and biochemical data analysis between the groups**

	**Non obese group**				**Obese group**				**p**
	**n**	**Mean ± SD**	**Median**	**Range**	**n**	**Mean ± SD**	**Median**	**Range**	
Age (years)	19	35.1 ± 5.9	35	24 – 49	23	37.9 ± 7.3	38	26 - 49	0.2172
Body mass index	19	25.5 ± 2.4	26.5	19.1 – 28.7	23	35.8 ± 4.3	35.7	30.1 – 44.0	<0.0001
Waist (cm)	19	91.4 ± 8.6	94.0	74.1 – 105.3	23	118.8 ± 12.9	115.2	96.2 – 141.9	<0.0001
Hips (cm)	19	97.3 ± 6.6	97.0	82.2 – 106.1	23	114.9 ± 83.3	114.2	98.1 – 136.4	<0.0001
Waist-to-hip ratio	19	0.92 ± 0.07	0.94	0.8 – 1.01	23	1.03 ± 0.06	1.03	0.91 – 1.17	<0.0001
Serum Glucose (mmol/L)	19	4.9 ± 0.4	4.9	3.8 – 5.3	23	5.4 ± 0.8	5.3	4.4 – 6.9	0.0071
Seminal Glucose (mmol/L)	19	1.87 ± 0.69	1.65	0.96 – 3.69	23	1.54 ± 0.38	1.69	0.69 – 1.99	0.0747
Serum Insulin (mIU/L)	19	5.5 ± 1.8	6.2	3.2 – 9.2	23	12.5 ± 5.8	12.4	4.3 – 32.0	<0.0001
Seminal Insulin* (mIU/L)	19	208.8 ± 98.2	162.5	128.7 – 439.4	23	517.6 ± 256.5	476	175.9 – 1060	<0.0001
Serum Leptin (ng/ml)	19	4.1 ± 2.4	4.5	1.4 – 8.7	23	8.8 ± 8.5	8.6	1.4 – 38.6	0.0187
Seminal Leptin (ng/ml)	19	5.6 ± 3.8	5.0	1.4 – 18.7	23	12.9 ± 9.1	12.5	1.4 – 34.3	0.0016

All statistical analysis done via Student *t*-test except * = Mann–Whitney tests.

Serum glucose was significantly increased in the Ob group compared to the nOb group. However, although mean concentrations of seminal glucose were slightly decreased in the Ob group, this did not reach statistical significance. Both serum and seminal insulin was significantly increased in the Ob groups compared to the nOb groups. A total of 9 serum and seminal leptin results were below the limit of detection (LOD). This included a total of three in the nOb group and six in the Ob group. As the lab reports did not supply the actual values for these LOD results, the substitution method of LOD/squareroot(2) was used for data analysis. Based on the included samples, serum and seminal leptin concentrations were higher in the Ob group. Details of these biochemical results for each group are provided in Table 2.

Various ratios between the biological results were determined as represented in Table 3. Insulin sensitivity was determined using the Quantitative Insulin Sensitivity Check Index (QUICKI), calculated from fasting blood glucose and insulin concentrations. The mean QUICKI (insulin sensitivity) was significantly lower in the Ob group compared to the nOb group. Serum and seminal glucose-to-insulin ratio were both significantly lower in the Ob group, reflecting greater increases in insulin concentrations as compared to glucose concentrations in both serum and semen. Based on a QUICKI score of < 0.357 being a definition for insulin resistance (as provided by the reference ranges supplied by Pathcare Laboratories, Bellville, South Africa), 61.9% of the cohort could be described as insulin resistance. In the nOb group, 15.8% were insulin resistance, whereas 100% of participants in the Ob group were diagnosed with insulin resistance (Table 1). Mean glucose concentrations were higher in the serum as compared to semen in both groups, with no difference between them. Although this ratio was slightly lower in the Ob group, this difference was not significant. The mean seminal-to-serum ratio for insulin was high in both the nOb (39.1 ± 17.0) and Ob groups (42.1 ± 15.0), although this was not statistically different. Although the mean seminal-to-serum ratio for leptin was higher in the Ob group (3.01 ± 3.29) compared to the nOb group (1.88 ± 1.28), this was not a significant difference.

**Table 3:** B**iochemical ratios based on serum and seminal assays between the group**

	**Non obese group**				**Obese group**				**P**
	**n**	**Mean ± SD**	**Median**	**Range**	**n**	**Mean ± SD**	**Median**	**Range**	
Serum QUICKI (Insulin sensitivity)	19	0.373 ± 0.02	0.364	0.340 – 0.407	23	0.329 ± 0.022	0.238	0.276 – 0.380	<0.0001
Serum glucose to insulin	19	0.95 ± 0.33	0.8	0.6 – 1.5	23	0.53 ± 0.31	0.4	0.2 – 1.3	<0.0001
Seminal glucose to insulin	19	0.012 ± 0.006	0.009	0.003 – 0.027	23	0.004 ± 0.002	0.004	0.001 – 0.01	<0.0001
Seminal to serum glucose	19	0.39 ± 0.18	0.34	0.19 – 0.97	23	0.29 ± 0.08	0.29	0.13 – 0.4	0.0834
Seminal to serum insulin	19	39.1 ± 17.0	40.0	21.7 – 91.4	23	42.1 ± 15.0	40.0	20.3 – 84.6	0.4529
Seminal to serum leptin	19	1.74 ± 1.21	1.01	0.74 – 4.18	17	2.49 ± 2.94	1.4	0.24 – 12.22	0.2447

All statistical analysis done using Mann–Whitney tests.

Detailed results of semen analyses for both groups are provided in Table 4. Sperm concentration and vitality were significantly lower in the Ob group. Although mean values were decreased on the Ob group compared to the nOb group, there were no significant differences between the groups for semen volume, total sperm count, progressive and total motility and normal sperm morphology. Percentage of sperm with abnormal mitochondrial membrane potential (MMP) and DNA fragmentation (DF) was significantly increased in the Ob group. Interestingly, the expected values for semen parameters in the nOb group were generally worse than expected for an otherwise healthy male cohort, particularly for motility, vitality and morphology. There are no studies on the local population in which to compare these results, and no data available in which to further discuss this observation.

**Table 4:** S**emen analysis between the groups**

	**Non obese group**				**Obese group**				**p**
	**n**	**Mean ± SD**	**Median**	**Range**	**n**	**Mean ± SD**	**Median**	**Range**	
Semen Volume (ml)	19	2.7 ± 1.0	2.7	1.2 – 5.5	23	2.5 ± 1.5	2.2	0.4 – 7.0	0.6217
Sperm conc. (10^6^/ml)	19	35.3 ± 16.7	34.0	8.8 – 72.4	23	23.7 ± 13.6	21.9	7.5 – 49.5	0.0145
Total sperm count (x10^6^)	19	96.5 ± 59.1	95.2	13.2 – 243.7	23	64.4 ± 58.8	47.3	3.7 – 247.5	0.0863
Progressive motility (%)	19	33.8 ± 16.2	33.7	0.0 – 59.5	23	24.5 ± 19.1	20.0	0.0 – 70.1	0.0986
Total motility (%)	19	52.2 ± 20.3	54.4	18.5 – 78.6	23	41.4 ± 21.6	42.2	1.1 – 74.9	0.1066
Vitality (%)	19	62.6 ± 18.1	60.0	29.0 – 92.0	23	45.0 ± 26.1	50.0	6.0 – 88.0	0.0172
Normal morphology (%)	19	2.57 ± 1.95	2.0	1.0 – 7.0	23	1.95 ± 1.22	2.0	0.0 – 5.0	0.2371
Abnormal MMP (%)	19	29.4 ± 13.4	24.0	15.0 – 58.0	23	57.7 ± 23.8	52.0	21.5 – 93.0	<0.0001
DNA Fragmentation (%)	19	17.3 ± 11.8	15.0	3.2 – 45.4	23	30.2 ± 18.6	29.5	5.0 – 83.5	0.0119

All statistical analysis done via Student *t*-test.

A relatively large percentage of participants had poor semen parameters according to WHO (2010) criteria; 26.2% with oligozoospermia, 42.9% with asthenozoospermia, 61.9% with necrozoospermia, 78.6% with teratozoospermia, 50% with increased percentage of sperm with abnormal mitochondrial membrane potential (MMP) and 42.9% with increased DNA fragmentation (DF) percentage. Although the Ob group had high percentage of participants presenting with all abnormal sperm parameters, only MMP and DF reached a statistical difference in incidence between the groups (Table 1). In addition, 21.4% of the cohort fulfilled the WHO (2010) definition of couple infertility with a non-significant increase in incidence in the Ob group (Table 1), although female factor infertility was not excluded in these cases.

**Figure 1:**
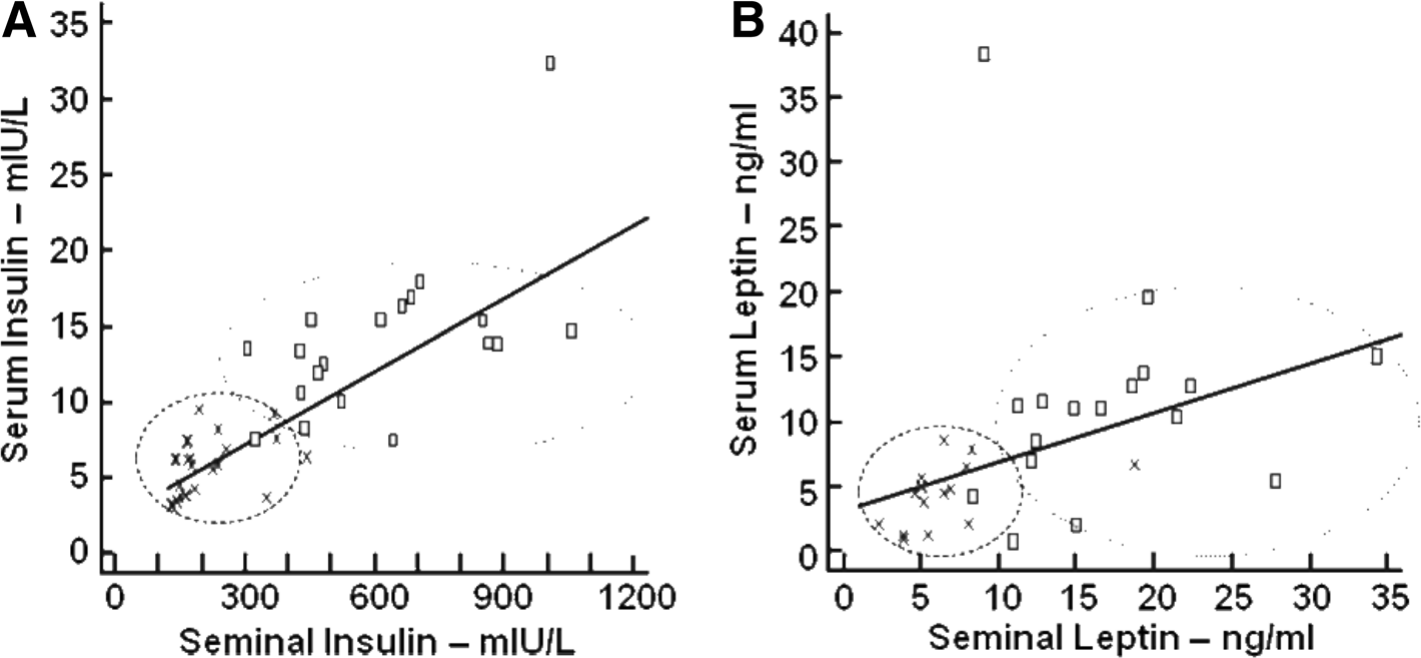
**Correlations between serum and seminal insulin (A) and leptin (B) concentrations within the cohort.** A significant correlation (r^2^ = 0.83) between serum and seminal insulin concentrations **(A)** was found; similarly, a significant correlation (r^2^ = 0.52) between serum and seminal leptin **(B)** concentrations was found. The figures show the distribution of the non-obese and obese groups, reflecting a clear increase in serum and seminal concentrations of both hormones with obesity. x = participants in the non-obese group (circled with the dash lines); □ = participants in the obese group (circled with the dotted line).

Due to the relatively small sample size within each group, correlations were assessed on the clinical and biochemical data of the entire cohort and not within each individual group. Although the QUICKI is included in the correlation assessments, no other ratios as provided in Table 3 were investigated for potential correlations. Correlations between the clinical and biochemical data were generally as expected, as were correlations for semen analysis parameters, and both sets of data are not shown. Correlations between the clinical, biochemical and semen analysis are provided in Table 5. Figure 1 shows the correlations between serum and seminal insulin (Figure 1A) and serum and seminal leptin (Figure 1B), illustrating clear differences between the two groups.

**Table 5:** Correlations between clinical, biochemical and semen analysis

		**BMI**	**WC**	**HC**	**WHR**	**Serum glucose**	**Seminal glucose**	**Serum insulin**	**Seminal insulin**	**Serum leptin**	**Seminal leptin**	**QUICKI**
Semen Volume	r^2^	NS	NS	NS	NS	NS	NS	NS	NS	NS	NS	NS
	P											
Sperm Concentration	r^2^	-0.362	-0.323	-0.311	NS	NS	NS	-0.311	-0.334	NS	NS	0.404
								0.0448	0.0307			0.0080
	P	0.0185	0.0370	0.0451								
Total Sperm Count	r^2^	-0.332	NS	NS	NS	NS	NS	-0.321	NS	NS	NS	0.400
												0.0086
	P	0.0315						0.0380				
Progressive Motility	r^2^	NS	NS	NS	NS	-0.318	NS	NS	NS	NS	NS	NS
						0.0401						
	P											
Total Motility	r^2^	NS	-0.324	NS	-0.323	-0.308	NS	NS	NS	NS	NS	NS
	P		0.0364		0.0366	0.0473						
Vitality	r^2^	-0.315	-0.400	-0.366	-0.320	NS	NS	NS	NS	NS	NS	NS
	P	0.0423	0.0087	0.0170	0.0390							
Morphology	r^2^	NS	NS	NS	NS	NS	NS	NS	NS	NS	NS	NS
	P											
MMP	r^2^	0.571	0.571	0.550	0.411	0.338	NS	0.390	0.358	NS	NS	-0.457
	P	0.0001	0.0001	0.0002	0.0068	0.0286		0.0107	0.0450			
												0.0024
DF	r^2^	0.396	0.415	0.306	0.415	NS	NS	NS	NS	NS	NS	NS
	P	0.0467	0.0063	0.0489	0.0063							

## Discussion

Both obesity and male factor infertility have coincidently been increasing globally over the last few decades [13]. Studies reporting on any relationship between obesity and male fertility have been generally inconsistent and inconclusive [5,12,14,15]. Obesity is associated with various metabolic changes, including glucose intolerance, insulin resistance (hyperinsulinaemia), hyperleptinaemia, chronic inflammation and, in males, hypogonadism [3-5]. Insulin and leptin are present in seminal fluid, modulating sperm function post ejaculation in addition to regulating male reproductive pathways centrally and peripherally [18-21]. Changes in serum concentrations of glucose, insulin and leptin may be associated with changes in seminal concentrations, previously unreported in the literature. Therefore, in addition to a potential negative influence of sperm function in obese males, seminal fluid quality may also be negatively influenced. This pilot study aimed to investigate seminal fluid concentrations of glucose, insulin and leptin in relation to serum concentrations and sperm function in 19 non-obese (nOb) and 23 obese (Ob) males.

### Obesity and semen parameters

The results generally agree with literature demonstrating that obesity has a negative impact on standard semen parameters, although other studies indicate no relationship [5;12;14;15]. This is particularly evident by decreased sperm concentration and vitality, with BMI correlating negatively with sperm concentration total sperm count (TSC) and vitality. Furthermore, WC and HC correlated negatively with sperm concentration and vitality, but not TSC. In addition, there were lower mean values for TSC, total and progressive motility and morphology in the Ob group, although these did not reach statistical significance. WC and WHR correlated negatively with total motility, however, there was no correlation between morphology and BMI, nor any other sperm parameter, in this study. Details of previous studies are available in the referenced reviews and meta-analyses [5,12,14,15].

Based on the WHO (2010) criteria for semen analysis [6], both groups had mean results above the recommended cut-off values for ejaculation volume (<1.5 ml), sperm concentration (<15 × 10^6^ million/ml) and TSC (<39 × 10^6^ million/ml). However, only the Ob group had mean percentages below the recommended cut-off values for total and progressive motility (<40% and < 32%, respectively) and vitality (<58%). Although the Ob group have higher percentages of the cohort presenting with oligozoospermia, asthenozoospermia and/or necrozoospermia, this difference was not significant (which may be due to the small sample size). Teratozoospermia (<4% normal morphology) was diagnosed in 78.6% of the cohort, and 68.4% of the nOB cohort and 86.9% of the Ob cohort. This high percentage in both groups may explain the lack of correlation between morphology and all other parameters in this study. Interestingly, 21.4% of the cohort had a history of recent couple infertility as defined by WHO (2010) [6]. However, it is important to note that no males or female partners had received any medical investigation for this complaint at the time of the clinical consultation. Although not statistically significant, a higher proportion of the Ob cohort (30.4%) reported this history, in contrast to the nOb group (10.6%). It must be clear, however, that this is defined as couple infertility and no further data on male or female factor infertility is available, and this should not be interpreted as male factor infertility percentages which were not established in the study. Furthermore, no males had consulted a medical or health care professional for infertility related complaints at the time of consultation.

Fewer studies have assessed the impact of obesity on DF and MMP. Both of these parameters were significantly increased in the Ob group compared to the nOb group. Furthermore, MMP and DF both correlated with BMI, WC, HC and WHR. The negative impact of BMI on DF and MMP confirms similar findings in previous studies [5,14,29]. A negative impact on MMP and DF in obesity appears to be a consistent variable in the literature when included for analysis. Damage to the sperm mitochondria function is suggested to negatively affect oxidative phosphorylation, reducing ATP synthesis and thus energy availability for motility [9]. MMP is negatively correlated with vitality and total and progressive motility in this study, supporting this well defined relationship. Although it can be hypothesised that obesity related phenomena, such as inflammation, may mediate damage to spermatozoa mitochondria and DNA integrity, the mechanisms of these relationships require further investigation. Serum and seminal insulin and serum glucose (but not seminal glucose) correlated with MMP and not DF, with a negative correlation between QUICKI and MMP.

### Serum and seminal insulin and leptin

The concept of insulin resistance, closely associated with abdominal obesity and increased WC, is used to describe the process whereby target tissues develop impaired sensitivity to the action of the hormone, particularly in adipose tissue, liver and skeletal muscle [30-32]. The QUICKI is a useful assessment of insulin sensitivity, correlating closely with Euglycaemic Hyperinsulinaemic Clamp (a gold standard assessment of insulin resistance) across a wide range of glucose and insulin concentrations [22]. The predominantly adipocyte-derived polypeptide hormone Leptin regulates body weight, appetite and energy expenditure via hypothalamic modulation, in addition to modulation of the immune, endocrine, metabolic and reproductive systems [32,33]. Leptin has been strongly associated with a role in the pathophysiology of obesity and metabolic syndrome, although this role has not been well understood or described [34]. Mean serum insulin and leptin was significantly increased in the Ob group as compared to the nOb group, with a significantly decreased QUICKI. As expected, serum insulin correlated positively with BMI, WC, HC and WHR. QUICKI correlated negatively with these same parameters. However, serum leptin did not correlate with these parameters as expected, possibly due to the relatively small sample size.

Various studies have assessed leptin concentrations in male serum, although an adequate reference range appears elusive. Raised serum leptin is also associated with sperm function changes in males [35,36]. The serum ranges of leptin in this study generally agree with concentrations found in healthy, obese and infertile male cohorts reported in the literature [35-38].

Insulin and leptin are important regulators of male reproduction via the HPT axis both centrally and peripherally [17], in addition to being present in human semen with important regulatory roles for sperm function and fertilisation [17-20]. Furthermore, both hormones are synthesised and secreted by ejaculated spermatozoa in an autocrine manner [18-20]. Exogenous addition of insulin and leptin to ejaculated semen has been shown to increase total motility, progressive motility, acrosome reaction and nitric oxide production in human spermatozoa [21]. Sertoli cells too have been shown to synthesise and secrete insulin [39].

The results show that obese males have significantly increased seminal insulin and leptin as compared to the nOb group. Strong correlations between serum and seminal insulin (r^2^ = 0.823), as well as serum and seminal leptin (r^2^ = 0.517), are reported. The negative correlations between both seminal insulin and leptin with BMI, WC, HC and WHR may indicate that these parameters are potential predictors of seminal changes of these hormones. Seminal insulin and leptin further correlated negatively with QUICKI (r^2^ = -0.782 and r^2^ = -0.311 respectively). These correlations indicate an important relationship between pathophysiological mediators of obesity and a decrease in male reproductive potential that requires further investigation.

The source of seminal insulin and leptin is not clear. The correlations between serum and semen concentrations may suggest that insulin and leptin in the semen gains access to the reproductive tract via the blood testes barrier (BTB), seminal vesicles or prostate. Since insulin and leptin are strongly associated with increases in obesity, it is plausible that there is a cross over from peripheral circulation to the reproductive tract. Insulin and insulin-like peptides in human semen have previously been suggested to be secreted by the seminal vesicles [40,41], and insulin appears to freely cross the BTB into the reproductive tract [42]. No apparent data on the source of leptin is available in the literature. In contrast, as insulin and leptin are synthesised and secreted in an autocrine fashion post-ejaculation, and Sertoli cells too secrete insulin within the testes [39], at least a local reproductive tract source of these hormones is also plausible. The source of these hormones in the reproductive tract requires further investigation.

Seminal and serum insulin was negatively correlated, and QUICKI positively correlated, with sperm concentration. The mechanisms for this are unclear. Type-1 diabetes mellitus, in which insulin is absent, is associated with a collapse of spermatogenesis and increased germ cell apoptosis [43]. Insulin resistance may mimic to some degree the loss of insulin in T1DM, and insulin resistance in Sertoli cells may hypothetically be associated with a decrease in spermatogenesis. As increased seminal insulin is associated with insulin resistance and abdominal obesity, increased insulin exposure during spermatogenesis may potentially develop insulin resistance in the Sertoli cells.

Although an acute *in vivo* increase in insulin and leptin exposure may increase motility and acrosome reaction in the spermatozoa [20,21], this study did not show significant correlations between seminal insulin and leptin with sperm motility and vitality. Increased insulin exposure during spermatogenesis may plausibly develop insulin resistance within the spermatozoa themselves. Evidence to support this hypothesis may be found in the intracellular molecular cascades associated with insulin receptor stimulation in these cells. Insulin, as well as leptin, exert its effect on spermatozoa via the PI3K/Akt intracelleular signalling pathway, leading to protein kinase B (PKB) phosphorylation [20], which may mediate beneficial effects on ejaculated spermatozoa [17]. This pathway ultimately increases cellular nitric oxide production [17;20]. In human tissues, this intracellular pathway is negatively influenced in insulin resistance [31]. Hypothetically, over the spermatogenic cycle, it is conceivable that spermatozoa may develop insulin resistance in a manner similar to other tissue cell via a breakdown of the PI3K/Akt intracelleular signalling pathway. This hypothesis would provide an explanation as to the potential negative association between increased seminal insulin and reduced motility of ejaculated sperm. Although Lampiao & du Plessis [21] found an increase in motility of ejaculated spermatozoa exposed to leptin, this was not found by Li and colleagues [44]. However, if this intracellular pathway does breakdown in spermatozoa, we would also expect to see a reduced or even negative correlation between seminal leptin and ejaculated sperm function. A model in which insulin and leptin resistance is induced in spermatozoa and Sertoli cells is required to further investigate this potential relationship.

Based on the seminal-to-serum insulin ratio, insulin was found to be highly concentrated in human semen in both groups. The concentration of insulin in human semen is somewhat supported by a limited number of studies across numerous groups, including fertile and infertile normoglycaemic subjects, carbohydrate intolerant subjects and excretory and secretary azoospermic subjects [42,45,46]. No plausible explanation for a physiological concentration of insulin in semen is apparent on a search of the literature. As a result of the insulin concentration, the seminal glucose-to-insulin ratio was lower than the serum glucose-to-insulin ratios. Both the serum and seminal glucose-to-insulin ratios where significantly lower in the Ob group due to a significant increase in serum and seminal insulin compared to a more subtle increase in serum glucose and a non-significant decrease in seminal glucose. This is likely due to the exclusion of participants with a high fasting glucose.

### Serum and seminal glucose

Glucose has been identified in human semen, with more than half the sugar consumed by ejaculated spermatozoa being in the form of glucose glycolysis [47,48]. Reports on normal concentrations vary widely, from 1.02 mmol/L – 5.7 mmol/L [47]. Seminal glucose concentration ranges in this cohort (0.69 – 3.69 mmol/L) were within these reported ranges. Although there was a lower mean in the Ob group compared to the nOb group, this did not reach statistical significance. A small sample size may be the reason for this not reaching statistical significance. Sampling indicates possible significance with n = 44 in the nOb group and n = 54 in the Ob group.

Serum glucose correlated positively serum insulin and negatively with QUICKI as would be expected, and further correlated negatively with spermatozoa motility and positively with MMP, indicating an association negative relationship between serum glucose and energy production in spermatozoa. Seminal glucose correlated negatively to BMI only. There was no correlation between serum and seminal glucose either.

Seminal glucose concentrations were relatively lower compared to serum levels, as indicated in the seminal-to-serum glucose ratio. This may be due to a tight control mechanism for glucose to pass from the peripheral circulation into the reproductive tract through the BTB, reducing glucose concentrations in order to optimally support and maintain spermatogensis. Testicular cells have glucose sensing machinary which enable them to react and adapt to hormonal fluctuations and counteract hyper- or hypoglycaemic events, as spermatogenesis maintainance *in vivo* is dependent on adequate glucose metabolism [49]. Glucose transport across the BTB is mediated by various glucose transport molecules (GLUT’s), such as GLUT1, GLUT3 and GLUT8, and are sensitive to various hormones (including insulin), inflammatory cytokines and growth factors [49]. With changes in glucose or insulin, glucose transport machinary adapts in order to maintain lactate production [49]. Insulin deprived Sertoli cells in culture show decreased glucose uptake via the BTB barrier [49]. Therefore, it may be biologically pausible that insulin resistance in the setting of obesity may be associated with a decrease in glucose uptake across the BTB. This is however hypothetical, and further research in the physiology and pathophysiology of the BTB in relation to glucose is required. However, there was a non-significant trend for seminal glucose to be decreased in the Ob group, whereas serum glucose was significantly increased, which may warrent further insight.

## Conclusions

Obesity is associated with increased serum and seminal insulin and leptin in a cohort of male participants. Hyperinsulinaemia and hyperleptinaemia associated with obesity may negatively impact reproductive function and fertility. Furthermore, insulin was highly concentrated in seminal fluid as compared to serum concentrations. The mechanisms associated with these findings, as well as the implications, require further investigations.

## Abbreviations

Akt: Akt kinase; BMI: Body mass index; BSA: Bovine serum albumin; BTB: Blood testes barrier; CVD: Cardiovascular disease; DF: DNA fragmentation; FSH: Follicular stimulating hormone; GLUT: Glucose transport molecules; GnRH: Gonadotropin releasing hormone; HC: Hip circumference; HTFM: Human tubular fluid medium; LH: Luteneizing hormone; MMP: Mitochondrial membrane potential; NS: Not significant (p > 0.05); nOb: Non-obese group; Ob: Obese group; PBS: Phosphate buffer solution; PI3K: Phosphoinositol-3-kinase; PKB: Protein kinase B; QUICKI: Quantitative insulin sensitivity check index; T1DM: Type-1 diabetes mellitus; T2DM: Type-2 diabetes mellitus; TSC: Total sperm count; TUNEL: Terminal deoxynucleotidyl transferase dUTP nick end labelling; WC: Waist circumference; WHO: World Health Organisation; WHR: waist-to-hip-ratio.

## Competing interests

The authors declare they have no competing interests at the time of publication.

## Authors’ contributions

KL conceived and contributed to the design of the study, recruitment of participants, collection of data, laboratory processing and various sample assays, performed the statistical analysis and drafted the manuscript. RM carried out the sperm morphology assessments and critically reviewed the manuscript. PB contributed to the design of the study and various serum and seminal assays**.** RH contributed to the design and coordination of the study, reviewed and contributed to the statistical analysis and critically reviewed and revised the manuscript. All authors read and approved the final manuscript.
